# HPV Oncoproteins and Mitochondrial Reprogramming: The Central Role of ROMO1 in Oxidative Stress and Metabolic Shifts

**DOI:** 10.3390/cells14201629

**Published:** 2025-10-19

**Authors:** Eva Tsoneva, Angel Yordanov

**Affiliations:** 1 Department of Reproductive Medicine, Specialized Hospital for Active Treatment of Obstetrics and Gynaecology Dr Shterev, 1330 Sofia, Bulgaria; dretsoneva@gmail.com; 2 Faculty of Medicine, Medical University Pleven, 5800 Pleven, Bulgaria; 3 Department of Gynaecological Oncology, Medical University Pleven, 5800 Pleven, Bulgaria

**Keywords:** ROMO1, ROS, high-risk human papillomaviruses, HPV-driven oncogenesis

## Abstract

High-risk human papillomaviruses (HPVs), particularly types 16 and 18, drive carcinogenesis by rewiring host metabolism and mitochondrial function. The oncoproteins E5, E6, and E7 collectively induce mitochondrial fragmentation, increase reactive oxygen species (ROS), and promote a metabolic shift from oxidative phosphorylation (OXPHOS) to glycolysis (the Warburg effect). A redox-sensitive mitochondrial protein, Reactive Oxygen Species Modulator 1 (ROMO1), has emerged as a key mediator of these processes. ROMO1 contributes to mitochondrial morphology, regulates ROS homeostasis, and interacts with key stress-response pathways. While ROMO1 is overexpressed in many cancers and correlates with poor prognosis, recent data suggest that HPV-associated cervical lesions exhibit a unique biphasic expression pattern, with high ROMO1 levels in early stages and reduced expression in advanced tumors. The underlying molecular mechanisms remain unclear, but may involve HPV genome integration, NF-κB suppression, or epigenetic silencing. Key mechanisms such as how HPV modulates ROMO1 expression and how this contributes to stage-dependent metabolic vulnerability remain incompletely understood. This review highlights the current understanding of how HPV oncoproteins impact mitochondrial structure and function, emphasizes the role of ROMO1 in this context, and compares findings with other cancer types. Although no ROMO1-targeted therapies currently exist, the protein may serve as a redox-sensitive biomarker and potential vulnerability in HPV-driven tumors. We propose that targeting mitochondrial fragmentation, ROS signaling, or metabolic reprogramming may offer new avenues for therapeutic intervention. Further research is needed to clarify ROMO1’s dual role in early vs. late-stage disease and to validate its relevance as a clinical target. Our review fills a gap in the current literature by being the first to systematically explore ROMO1’s contribution to HPV-induced mitochondrial dysfunction and metabolic rewiring, and we outline research priorities for future studies.

## 1. Introduction

Papillomaviruses are a large family of small, double-stranded DNA viruses, which can be divided into five genera (α, β, γ, µ and ν). The Alpha and Beta genera are the ones that cause health problems in humans and are therefore the most investigated [1]. α-HPVs (infect basal layer of mucosal epithelial cells) are divided into high-risk (HR) types, with oncogenic potential, and low-risk (LR) types, which typically lead to benign, self-limiting warts [1]. High-risk α-HPVs (such as HPV16 and HPV18) encode two major oncoproteins, E6 and E7, and a minor oncoprotein E5, which work together to induce cellular transformation [1]. E6 and E7 are well known for inactivating p53 and pRb tumor suppressors, respectively [2]. In addition to pushing the cell into uncontrolled growth, these viral proteins also reshape how the cell manages its metabolism and mitochondrial activity. HPV-positive cancers often exhibit metabolic reprogramming: they tend to favor aerobic glycolysis over OXPHOS, even when oxygen is sufficient [3]. This metabolic shift (the Warburg effect) provides rapidly dividing cells with biosynthetic precursors at the expense of ATP efficiency [4]. While the metabolic reprogramming associated with HPV infection has been reviewed previously, none of the existing literature has focused specifically on the role of the mitochondrial protein ROMO1 (Reactive Oxygen Species Modulator 1). ROMO1 is a redox-sensitive inner mitochondrial membrane protein that regulates ROS homeostasis and mitochondrial dynamics. Dysregulation of ROMO1 has been implicated in a variety of cancers, but its specific involvement in HPV-driven malignancy has not been critically evaluated. This review aims to fill this knowledge gap by systematically examining how HPV oncoproteins alter mitochondrial structure and energy metabolism, with a focus on ROMO1’s modulatory role. We highlight key questions that remain unanswered, such as how HPV may regulate ROMO1 expression, whether this regulation is subtype- or stage-specific, and what therapeutic implications may arise from ROMO1 dysregulation. We also propose that ROMO1 represents a potential stage-specific biomarker and therapeutic target in HPV-associated cancers. The sections that follow explore HPV’s effects on central carbon metabolism, mitochondrial dynamics, and ROS regulation, culminating in a focused discussion on ROMO1 as a critical node in this virus–host interaction.

## 2. Clinical Background and Rationale

The impetus for this review emerged from our own clinical research on ROMO1 expression in cervical cancer. In an initial cohort of 75 patients with confirmed cervical carcinoma, we observed an interesting pattern: ROMO1 expression was highest in early-stage tumors and progressively decreased as the disease advanced. This finding contrasted with published reports in non-HPV-related cancers, where ROMO1 is typically upregulated in advanced stages and correlates with poor prognosis. Motivated by this unexpected result, we expanded our study to include a larger patient cohort- including healthy cervical tissue, patients with cervical intraepithelial neoplasia (CIN), and invasive cervical cancer. Our expanded, yet to be published, dataset confirmed the biphasic expression of ROMO1 in HPV-associated disease. These observations led us to hypothesize that the viral etiology of cervical cancer may uniquely modulate ROMO1 regulation, distinguishing it from other malignancies. This review was therefore conceived to examine the mechanistic links between HPV oncoproteins, mitochondrial dynamics, oxidative stress, and ROMO1 function—an area that remains largely unexplored.

## 3. Scope of This Review

Previous reviews have broadly examined HPV-induced changes in mitochondrial function and metabolism [3,4]. However, they have not explored the role of ROMO1 in the context of HPV infection. This review addresses that gap by examining recent findings on ROMO1 regulation, its interaction with mitochondrial dynamics, and its potential role as a modulator of HPV-induced metabolic changes. We also highlight our own emerging data, suggesting that ROMO1 expression is biphasic in cervical cancer—a pattern that differs from other tumor types and may reflect virus-specific regulation.

## 4. Mechanisms of HPV-Mediated Mitochondrial Metabolic Reprogramming

At the mechanistic level, HPV oncoproteins stimulate glycolytic pathways while suppressing OXPHOS. E6 plays a central role by promoting the degradation of p53 through its interaction with the E6AP ubiquitin ligase, thereby eliminating p53’s normal restraint on glycolysis. Under physiological conditions, p53 supports oxidative metabolism by inducing TIGAR (TP53-induced glycolysis and apoptosis regulator) and SCO2 (cytochrome c oxidase assembly factor), both of which enhance OXPHOS and limit glycolytic flux [4]. E6-mediated p53 loss thus leads to reduced SCO2 and TIGAR, tilting the balance toward glycolysis [4]. In addition, high-risk E6 can stabilize hypoxia-inducible factor 1α (HIF-1α) by blocking its VHL-mediated degradation, thereby enhancing HIF-1–driven glycolytic gene expression [4]. E6 also activates key metabolic regulators like pyruvate kinase M2 (PKM2), PI3K/Akt, and mTOR, all of which favor glucose uptake and lactate production [4]. At the same time, E7 contributes to metabolic reprogramming by promoting cell-cycle progression through pRb/E2F inactivation and by enhancing HIF-1α activity. The loss of Rb function releases E2F transcription factors, which in turn upregulate a broad set of glycolytic enzymes and mitochondrial biogenesis genes, fostering a metabolic environment that supports malignant growth [4].

**Figure 1:**
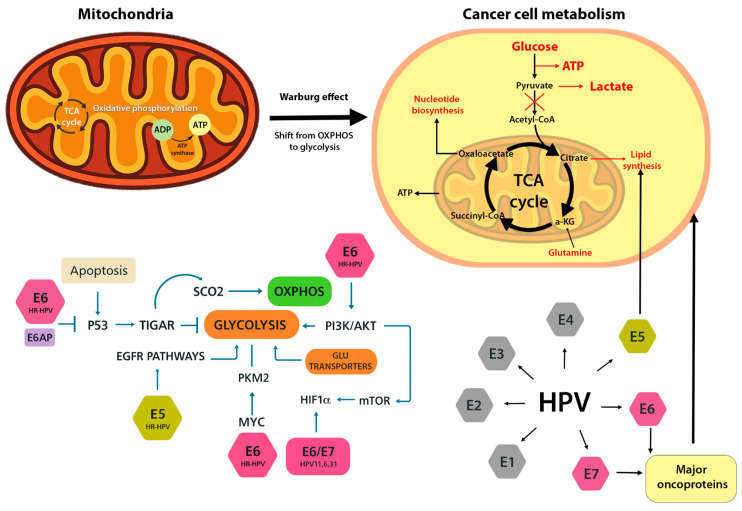
Metabolic reprogramming induced by HPV oncoproteins. HPV E6 and E7 promote a shift from oxidative phosphorylation (OXPHOS) to aerobic glycolysis (Warburg effect). E6 suppresses p53 and activates HIF-1α, upregulating glycolytic enzymes and glucose transporters. E7 disrupts pRb, enhances E2F activity, and stimulates mitochondrial biogenesis. E5 supports these changes via PI3K/Akt signaling. Together, these proteins rewire host cell metabolism to support proliferation and survival.

Although E5 alone is not sufficient to drive cellular transformation, it plays a supportive role in establishing the Warburg effect. Localized mainly to endosomal and Golgi membranes, E5 can activate growth factor signaling cascades. For example, HPV16 E5 enhances epidermal growth factor receptor (EGFR/ErbB) signaling and the downstream PI3K–Akt pathway, thereby promoting cell survival while increasing the expression of glucose transporters and glycolytic enzymes [1]. In concert, E5, E6, and E7 increase glucose uptake (via GLUT1/4 and SGLT transporters), elevate glycolytic flux, and suppress mitochondrial oxidative metabolism [4]. As a result, even in the presence of oxygen, HPV-transformed cells favor glucose fermentation to lactate, generating biosynthetic precursors needed for rapid proliferation. (Figure 1).

Importantly, these metabolic changes are closely linked to increased ROS production and oxidative stress. Mitochondria represent the primary source of ROS in cells, and when oxidative phosphorylation is impaired in favor of glycolysis, electron transport chain inefficiency often results in ROS accumulation [2]. HPV E6 has been shown to elevate intracellular ROS and oxidative stress markers. [2] For instance, expression of HPV16 or HPV18 E6 leads to higher steady-state ROS, depletion of glutathione (GSH), and a more oxidized redox balance [2]. This oxidative stress promotes DNA damage and genomic instability, thereby driving carcinogenesis [2]. E7 also contributes indirectly: by enforcing unscheduled S-phase entry and stimulating mitochondrial biogenesis, E7-infected cells can exhibit heightened mitochondrial activity without adequate quality control, further increasing ROS. Consistent with this, HPV-positive head and neck cancer cells display greater mitochondrial metabolism and ROS generation compared to HPV-negative cancers [2]. Collectively, high-risk HPV oncoproteins not only reprogram energy metabolism from OXPHOS to glycolysis, but also amplify ROS production, establishing a cellular environment highly favorable for malignant transformation.

## 5. Mitochondrial Morphology Changes in HPV Infection: Fission over Fusion

In healthy cells, mitochondrial networks are maintained through a continuous cycle of fission and fusion, processes essential for functional integrity [5]. This balance is regulated by key proteins, with Drp1 (dynamin-related protein 1) driving fission, and OPA1 together with MFN1 and MFN2 mediating fusion [5,6]. Disruption of this equilibrium is a hallmark of cancer and is often accompanied by metabolic reprogramming. HPV oncoproteins have been shown to disturb mitochondrial dynamics, generally shifting the balance toward excessive fission and fragmented mitochondria [3]. Such fragmented organelles are typically less efficient at oxidative phosphorylation (OXPHOS) and produce more ROS, consistent with HPV-induced mitochondrial phenotypes [7].

**Figure 2:**
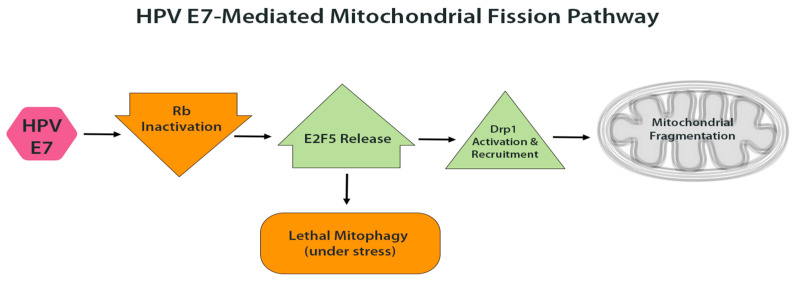
HPV E7-mediated mitochondrial fission. E7 inactivates pRb, releasing E2F5, which activates Drp1 and promotes its translocation to the mitochondrial outer membrane. This leads to increased mitochondrial fission and fragmentation. Under therapeutic stress, such fragmentation can trigger lethal mitophagy and sensitize cells to apoptosis.

An interesting example is the effect of HPV E7 on Drp1-mediated fission. In addition to promoting cell cycle progression through Rb inactivation, E7 also influences mitochondrial dynamics. Studies in HPV-positive head and neck cancer models demonstrated that E7 enhances Drp1 activation and translocation to the mitochondrial outer membrane, leading to excessive fission [8]. Mechanistically, the loss of Rb releases the transcription factor E2F5, which associates with Drp1 and serves as a scaffold to promote its activation and recruitment [8]. The resulting mitochondrial fragmentation can, under stress conditions such as chemotherapy, trigger ‘lethal mitophagy,’ a form of cell death in which highly fragmented mitochondria are selectively degraded by autophagy [8]. This mechanism may partly underlie the improved response of HPV-positive tumors to DNA-damaging therapies, as E7 sensitizes mitochondria to mitophagy-induced death under stress [8]. Even in the absence of external insults, however, E7-driven mitochondrial fragmentation likely contributes to baseline mitochondrial dysfunction in HPV-infected cells (Figure 2)

**Figure 3:**
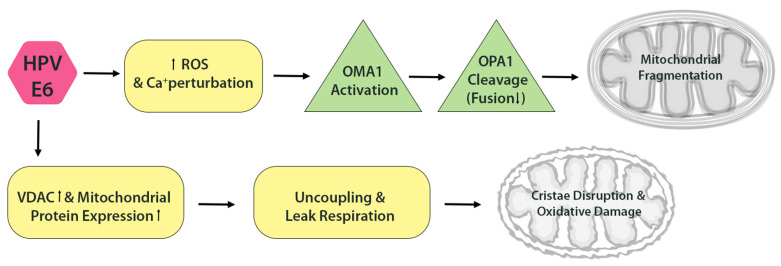
HPV E6 disrupts mitochondrial dynamics. E6 elevates ROS and induces OPA1 cleavage via activation of the OMA1 protease, impairing mitochondrial fusion. E6 also promotes mitochondrial uncoupling and proton leak, leading to cristae disorganization and further oxidative damage.

HPV E6 may also influence mitochondrial dynamics, albeit more indirectly. By elevating intracellular ROS, E6 can activate stress-responsive proteases such as OMA1, which cleave OPA1-the inner membrane fusion protein-thereby impairing mitochondrial fusion and promoting fragmentation. Both excessive ROS and calcium dysregulation, features observed in HPV-expressing cells, are known triggers of OPA1 cleavage. Interestingly, a 2019 study reported that HPV16 and HPV18 E6 increase the expression of voltage-dependent anion channel (VDAC) and other mitochondrial proteins, yet paradoxically induce mitochondrial uncoupling and ‘leak’ respiration [2]. This uncoupling, essentially a proton leak across the inner membrane, results in swollen or structurally abnormal mitochondria. Indeed, cells expressing E6 exhibit evidence of decoupled respiration and oxidative damage [2], changes that are frequently associated with disrupted cristae architecture and enhanced mitochondrial fragmentation (Figure 3).

Even the viral pro-oncogenic protein E2, despite often being lost upon viral genome integration, can influence mitochondrial function. HPV18 E2 has been shown to physically interact with respiratory chain Complexes III, IV, and ATP synthase, leading to disruption of cristae architecture, increased mitochondrial ROS (mROS) release, and stabilization of HIF-1α [3]. This stabilization promotes the glycolytic shift through HIF-1 targets such as PDK1 and CAIX [3]. Similarly, overexpression of HPV16 E2 induces mitochondrial dysfunction, ROS accumulation, and, in certain models, even apoptosis [3]. Together, these findings highlight that multiple HPV proteins, both early and late, converge on disturbing mitochondrial integrity.

Overall, HPV-infected cells characteristically display smaller, fragmented mitochondria rather than elongated networks. Viral oncoproteins bias this dynamic balance toward fission, driven by Drp1 activation through E7 [8] and potentially reinforced by E6-induced OPA1 cleavage under stress conditions. Fusion is simultaneously impaired, tipping the network toward a fragmented phenotype. Functionally, this morphology is coupled to metabolic reprogramming: fragmented mitochondria yield lower ATP per unit of oxygen consumed and generate more ROS, thereby reinforcing the Warburg effect while sustaining chronic oxidative stress [9].

Interestingly, mitochondrial fragmentation may also support viral persistence by modulating apoptosis. Smaller mitochondria are more prone to releasing cytochrome c and other apoptogenic factors, but HPV oncoproteins counteract this through anti-apoptotic mechanisms- for example, E6 degrades p53 and prevents Bax activation, thereby blocking cytochrome c release [1]. This suggests a fine-tuned viral strategy: enforcing sufficient mitochondrial fragmentation to reshape metabolism and signaling, while simultaneously inhibiting apoptosis to preserve host cell viability. Such reprogramming creates a precarious balance that fosters persistent infection and oncogenic progression. Although mitochondrial fragmentation and metabolic reprogramming co-occur in HPV-infected cells, whether one causes the other remains unproven. Future studies using Drp1 inhibitors such as Mdivi-1 could clarify whether mitochondrial fragmentation actively drives the Warburg effect or merely accompanies it. Importantly, this altered mitochondrial state can also become a vulnerability, as it may sensitize HPV-positive cells to oxidative damage or trigger lethal mitophagy under therapeutic stress [8].

## 6. ROMO1: A Mitochondrial Redox Sensor Hijacked by HPV

Among the host factors that intersect with HPV-driven mitochondrial alterations, Reactive Oxygen Species Modulator 1 (ROMO1) has emerged as a critical player. ROMO1 is a small, 79-amino-acid protein localized to the inner mitochondrial membrane (IMM), where it contributes to both ROS regulation and mitochondrial dynamics [10]. Under physiological conditions, ROMO1 functions as a redox sensor through its multiple cysteine residues, which undergo reversible disulfide bond formation in response to ROS [10]. Recent biochemical studies have shown that ROMO1 can directly scavenge H_2_O_2_, protecting protein thiols from irreversible oxidation. Through oxidative oligomerization, ROMO1 buffers excess ROS and shields IMM enzymes from damage [10]. This antioxidant role is particularly important given that the IMM is the primary site of ROS generation from the electron transport chain.

Beyond redox control, ROMO1 also regulates mitochondrial morphology. It is required for the import of the protease YME1L, which processes the fusion protein OPA1, thereby influencing mitochondrial inner membrane fusion [10]. A seminal study by Yordanov et al. identified ROMO1 as an “essential redox-dependent regulator of mitochondrial dynamics” [11]. Knockdown of ROMO1 results in abnormal mitochondrial morphology, ranging from elongated hyperfused networks to dysregulated fragmentation, depending on the cellular context [11]. Thus, ROMO1 links cellular redox state with mitochondrial shape and functional integrity.

In HPV-infected cells, ROMO1 expression appears to be dynamically regulated. An immunohistochemical study in cervical cancer (CC) patients found significantly higher ROMO1 expression in early-stage tumors (FIGO I) compared to advanced stages (FIGO II/III) [12]. Early lesions, typically characterized by episomal HPV and strong E6/E7 expression-exhibited robust ROMO1 staining, while more invasive cancers with integrated HPV genomes showed diminished ROMO1 [12]. This pattern suggests that ROMO1 may be upregulated initially to buffer HPV-induced oxidative stress, but downregulated later as the tumor progresses and accumulates additional mutations. This raises the possibility that ROMO1 modulation may contribute to tumor progression.

**Figure 4:**
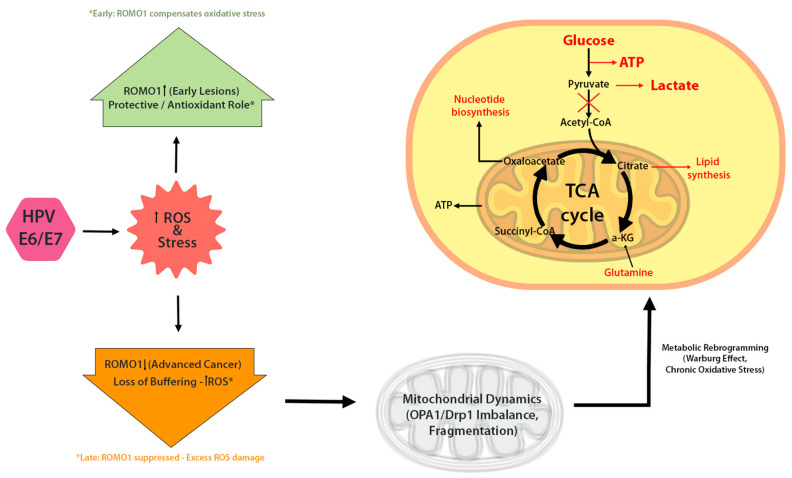
ROMO1 in HPV-driven mitochondrial reprogramming. Early HPV infection upregulates ROMO1 in response to oxidative stress, helping maintain redox homeostasis. In advanced stages, ROMO1 is often downregulated, resulting in elevated ROS, mitochondrial dysfunction, and increased DNA damage. ROMO1 may act as a key modulator of mitochondrial morphology and function in HPV-positive cancers.

This dynamic mirrors ROMO1’s dual role in cancer biology, which appears to be highly context-dependent. In some cancer types, elevated ROMO1 expression correlates with enhanced proliferation and invasion, likely through the generation of sub-lethal ROS levels that activate mitogenic pathways such as ERK and NF-κB. For example, in colorectal cancer and hepatocellular carcinoma, ROMO1 overexpression has been linked to tumor progression, increased migration, and poor prognosis [13,14]. Conversely, in other contexts, ROMO1 seems to function protectively by buffering oxidative stress and maintaining mitochondrial integrity. For instance, ROMO1 is necessary for OPA1-dependent cristae maintenance in mammalian cells, overexpression of ROMO1 in murine tissues protects the mitochondrial cysteinome from oxidative damage, and in porcine embryos, ROMO1 knockdown causes ROS accumulation, mitochondrial dysfunction, and apoptosis [15,16]. In HPV-positive cells, the scenario may be biphasic: during early infection, ROMO1 is upregulated to mitigate E6/E7-induced ROS, whereas in advanced stages, its expression may be suppressed, exacerbating oxidative damage and promoting tumor progression.(Figure 4) This dual behavior raises the possibility that ROMO1 could serve as a biomarker or therapeutic target in HPV-associated and other malignancies, but this hypothesis requires functional validation. Beyond HPV-associated cancers, ROMO1 plays a significant role in the biology of other tumor types. In hepatocellular carcinoma, colorectal cancer, and breast cancer, overexpression of ROMO1 has been associated with enhanced tumor cell proliferation, migration, and invasion, often linked to ROS-mediated activation of MAPK and NF-κB pathways [1,6,7]. Conversely, while direct evidence in lung and gastric cancers is limited, some studies suggest that ROMO1 depletion may impair mitochondrial function or increase susceptibility to oxidative damage [7,8]. The absence of functional studies specifically addressing ROMO1 silencing in these cancer types highlights an important gap in our understanding and represents a promising avenue for future investigation. These findings suggest that ROMO1 may act as either a tumor promoter or suppressor depending on the redox environment and cellular context. Exploring this dichotomy across cancer types can inform how HPV co-opts ROMO1 for its own oncogenic advantage.

Mechanistically, how HPV regulates ROMO1 remains unclear. One possibility involves NF-κB, a transcription factor that can induce ROMO1 under stress conditions [17,18]. Although the exact mechanism by which HPV regulates ROMO1 remains unclear, potential pathways include E6/E7-mediated suppression of NF-κB, p53 inactivation, and epigenetic silencing, all of which have been implicated in HPV pathogenesis. However, these regulatory mechanisms remain speculative in the context of ROMO1 and warrant experimental validation. HPV16 E6/E7 are known to suppress NF-κB activity to evade immune responses [19,20], which may secondarily reduce ROMO1 expression. Additional mechanisms, such as p53 inactivation or epigenetic silencing, may also contribute.

Recent evidence underscores ROMO1’s central role in mitochondrial health. A 2025 Nature Communications study demonstrated that ROMO1 overexpression protects against mitochondrial oxidative damage and age-related dysfunction [10]. While this review focuses primarily on HPV16 and HPV18, which are the most studied high-risk types, other oncogenic subtypes such as HPV31, HPV33, and HPV45 may exert distinct effects on mitochondrial dynamics and ROMO1 regulation. These differences remain poorly characterized and deserve further study to determine whether mitochondrial reprogramming is subtype-specific Mice overexpressing ROMO1 displayed a reductive proteomic shift (maintaining cysteines in a reduced state), improved respiratory capacity, and resistance to permeability transition pore opening. In cell models, ROMO1 upregulation enhanced OXPHOS, stabilized calcium uptake, and reduced ROS accumulation [10]. Thus, HPV-driven ROMO1 insufficiency would be expected to impair energy metabolism, elevate oxidative stress, and amplify the Warburg phenotype.

From a therapeutic standpoint, ROMO1 represents a potential biomarker and target. Its expression may indicate disease stage, may indicate whether the disease is HPV triggered, or oxidative stress burden in HPV-associated tumors. Strategies to restore ROMO1 could normalize mitochondrial dynamics and mitigate ROS, while conversely, inhibiting ROMO1 might push HPV-positive cells toward catastrophic oxidative stress-potentially in synergy with pro-oxidant therapies. Such interventions require caution given ROMO1’s essential roles in normal physiology, but they highlight the broader principle that understanding HPV’s disruption of mitochondrial biology can uncover novel therapeutic opportunities.

## 7. Integrative Model: From HPV Oncoproteins to Mitochondrial Dysfunction via ROMO1

**Figure 5:**
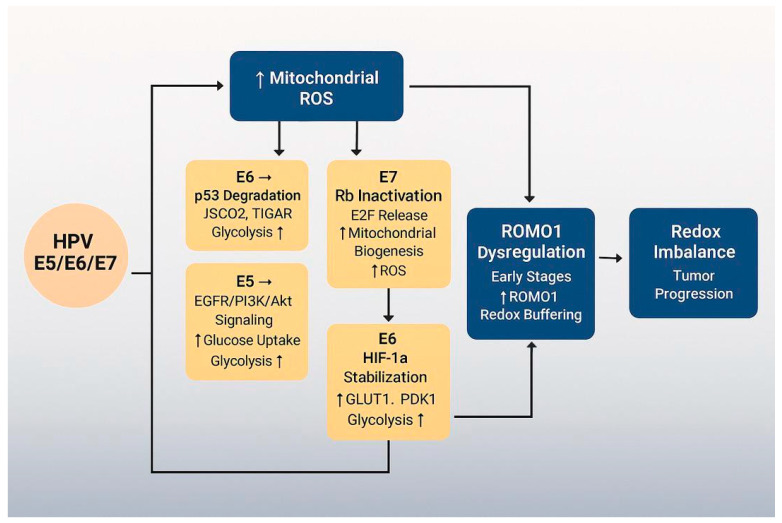
Integrative model of HPV-mediated mitochondrial dysfunction.

To enhance conceptual clarity, we propose a unifying model that links the distinct mitochondrial and metabolic changes described in this review (Figure 5). This pathway begins with HPV oncoproteins E5, E6, and E7, which reprogram host cell metabolism by promoting aerobic glycolysis and suppressing oxidative phosphorylation. These metabolic shifts are accompanied by increased ROS production, driven by mitochondrial dysfunction and loss of redox balance. In parallel, HPV oncoproteins disrupt mitochondrial dynamics, tipping the balance toward fragmentation via Drp1 activation and OPA1 inhibition. These changes not only impair energy production but also sensitize mitochondria to oxidative stress and mitophagy.

At the center of this cascade lies ROMO1, a redox-sensitive protein that links mitochondrial morphology to oxidative stress regulation. ROMO1 expression is initially upregulated in early HPV infection, likely as a compensatory response to rising ROS, but becomes suppressed as cancer progresses—potentially due to HPV genome integration or epigenetic silencing.

This integrative sequence—HPV oncoproteins → metabolic reprogramming → mitochondrial fragmentation → ROMO1 modulation → redox imbalance and tumor progression—captures the interplay between viral transformation, mitochondrial structure and function, and disease evolution.

By placing ROMO1 within this framework, we identify it as both a mediator and potential marker of stage-specific vulnerabilities in HPV-associated cancers. Future research should test this model in functional studies and assess whether targeting nodes such as Drp1 or ROMO1 can yield therapeutic benefit.

This diagram illustrates the proposed sequence of events in HPV-associated mitochondrial reprogramming. HPV oncoproteins E5, E6, and E7 initiate metabolic reprogramming by shifting cellular energy production from oxidative phosphorylation (OXPHOS) to aerobic glycolysis (Warburg effect). This shift promotes increased mitochondrial ROS (mROS) levels, redox imbalance, and mitochondrial dysfunction. ROMO1, a redox-sensitive mitochondrial protein, plays a dual role—initially buffering oxidative stress in early stages but potentially becoming downregulated as cancer progresses. The cumulative effect of viral oncoprotein activity and ROMO1 dysregulation contributes to tumor progression and creates a redox-vulnerable state that may be exploited therapeutically.

## 8. Conclusions and Therapeutic Implications

### 8.1. Summary of Current Understanding

HPV oncoproteins E5, E6, and E7 fundamentally rewire the host cell’s powerhouses-the mitochondria. They drive a shift away from efficient oxidative phosphorylation toward glycolysis, a less efficient but proliferation-favoring pathway that underlies the Warburg metabolism common in cancers. In doing so, they generate a state of chronic oxidative stress, which fuels genetic instability and accelerates tumor progression. A critical aspect of this reprogramming is the alteration of mitochondrial structure: HPV-infected cells frequently harbor fragmented mitochondria with disrupted cristae. These structural changes are not simply bystanders; they are functionally coupled to metabolic remodeling and influence whether a cell adapts under stress or succumbs to death-particularly evident in E7–Drp1–driven mitophagy during therapy. Emerging evidence suggests that HPV may regulate ROMO1 expression during infection and transformation, contributing to stage-dependent differences in redox stress and metabolic vulnerability.

### 8.2. Knowledge Gaps

Although ROMO1 has been studied in the context of other cancers, its mechanistic regulation by HPV oncoproteins remains unclear. It is unknown whether viral suppression of NF-κB, p53 inactivation, or epigenetic silencing are responsible for reduced ROMO1 expression in late-stage tumors. In addition, the interplay between mitochondrial fragmentation and metabolic reprogramming in HPV-positive cells has not been causally demonstrated. Furthermore, differences in ROMO1 dynamics across high-risk HPV subtypes (e.g., HPV16 vs. HPV31/45) are largely unexplored.

### 8.3. Clinical Implications and Limitations

These insights open new therapeutic opportunities. Approaches that normalize mitochondrial dynamics-for instance, Drp1 inhibitors to limit excessive fission or compounds that promote fusion-might restore healthier networks and resensitize HPV+ cells to apoptosis “[21,22,23]”. Conversely, strategies that exploit mitochondrial fragility, such as pro-oxidant therapies [24,25] or metabolic inhibitors [26,27,28] could selectively eliminate HPV-infected cells already vulnerable to redox imbalance. (Table 1) Importantly, the unique metabolic profiles of HPV+ tumors (sometimes OXPHOS-reliant, other times hyper-glycolytic) suggest that stage- or subtype-specific interventions may be possible. ROMO1 shows potential as a biomarker for disease stage and mitochondrial dysfunction in HPV-associated lesions, particularly cervical cancer. Its biphasic expression pattern—high in early lesions and diminished in advanced cancers—may help stratify patients or inform prognosis. This pattern may be explained by HPV genome integration, which disrupts E6/E7 expression and redox regulation, or by host-driven epigenetic silencing. Alternatively, tumor progression may shift metabolic needs, reducing the necessity for ROMO1-mediated ROS control. However, the clinical utility of ROMO1 is limited by a lack of large, reproducible cohort studies and a poor understanding of its upstream regulators. Moreover, given its essential role in normal mitochondrial physiology, targeting ROMO1 directly may carry toxicity risks unless precisely modulated.

Table 1 summarizes experimental and clinically investigated therapeutic agents relevant to pathways discussed in the review, including mitochondrial fission, oxidative stress, metabolic signaling, and epigenetic regulation. Each entry includes the proposed mechanism of action, relevance to ROMO1 and clinical trial information where applicable. These therapies are included to illustrate potential intervention points; however, most remain in the experimental stage and are not specific to ROMO1. Further research is needed to assess their applicability in HPV-positive cancers.

### 8.4. Future Research Directions

Future studies should investigate:

- How HPV regulates ROMO1 expression at the transcriptional, epigenetic, or post-translational level
- Whether ROMO1 modulation is HPV-type specific
- The functional consequences of ROMO1 knockdown or overexpression in HPV+ cell models and animal systems
- The potential of combining ROMO1-targeted interventions with Drp1 inhibitors, pro-oxidant therapies, or metabolic reprogramming agents

Understanding these mechanisms could uncover novel therapeutic strategies that exploit mitochondrial vulnerabilities in HPV-driven cancers.

## Figures and Tables

**Table 1:** Therapeutic Strategies Related to ROMO1 and Mitochondrial Redox Modulation.

Therapeutic Strategy	Agent/Drug	Target/Mechanism	Relevance to ROMO1	Evidence Type	Reference
Drp1 inhibition	Mdivi-1	Inhibits mitochondrial fission, promotes fusion	May stabilize mitochondria and reduce ROS load	Preclinical (in vitro, in vivo)	Bordt EA et al. (2017) [29]
Pro-oxidant therapy	Elesclomol	Elevates ROS beyond threshold to induce cell death	HPV+ cells with reduced ROMO1 may be sensitized	Conceptual/Preclinical	Kirshner JR et al. (2008) [30]
Antioxidants	N-acetylcysteine (NAC)	Replenishes glutathione, reduces intracellular ROS	May restore redox balance in early HPV-induced lesions	Experimental (cell models)	Samuni Y et al. (2013) [31]
NF-κB modulation	Parthenolide (experimental)	Restores transcriptional regulation of ROMO1	NF-κB may be suppressed by HPV E6/E7	Conceptual	Kwok et al., 2001 [32]
ROMO1-targeted modulation	(No current drug)	Hypothetical-restoring/modulating ROMO1 function	Hypothetical-restoring/modulating ROMO1 function	Theoretical	This review

## Data Availability

The authors declare that all related data are available from the corresponding author upon reasonable request.

## References

[1] Skelin J., Sabol I., Tomaić V. (2022). Do or Die: HPV E5, E6 and E7 in Cell Death Evasion. Pathogens.

[2] Cruz-Gregorio A., Aranda-Rivera A.K., Aparicio-Trejo O.E., Coronado-Martínez I., Pedraza-Chaverri J., Lizano M. (2019). E6 Oncoproteins from High-Risk Human Papillomavirus Induce Mitochondrial Metabolism in a Head and Neck Squamous Cell Carcinoma Model. Biomolecules.

[3] Cruz-Gregorio A., Aranda-Rivera A.K., Roviello G.N., Pedraza-Chaverri J. (2023). Targeting Mitochondrial Therapy in the Regulation of HPV Infection and HPV-Related Cancers. Pathogens.

[4] Martínez-Ramírez I., Carrillo-García A., Contreras-Paredes A., Ortiz-Sánchez E., Cruz-Gregorio A., Lizano M. (2018). Regulation of Cellular Metabolism by High-Risk Human Papillomaviruses. Int. J. Mol. Sci..

[5] Chan D.C. (2012). Fusion and fission: Interlinked processes critical for mitochondrial health. Annu. Rev. Genet..

[6] Tilokani L., Nagashima S., Paupe V., Prudent J. (2018). Mitochondrial dynamics: Overview of molecular mechanisms. Essays Biochem..

[7] Westermann B. (2012). Bioenergetic role of mitochondrial fusion and fission. Biochim. Biophys. Acta..

[8] Thomas R.J., Oleinik N., Panneer Selvam S., Vaena S.G., Dany M., Nganga R.N., Depalma R., Baron K.D., Kim J., Szulc Z.M. (2017). HPV/E7 induces chemotherapy-mediated tumor suppression by ceramide-dependent mitophagy. EMBO Mol. Med..

[9] Kanithi M., Junapudi S., Shah S.I., Matta Reddy A., Ullah G., Chidipi B. (2022). Alterations of Mitochondrial Network by Cigarette Smoking and E-Cigarette Vaping. Cells.

[10] Xu F., Huang H., Peng K., Jian C., Wu H., Jing Z., Qiu S., Chen Y., Liu K., Fu L. (2025). ROMO1 overexpression protects the mitochondrial cysteinome from oxidations in aging. Nat. Commun..

[11] Yordanov A., Tsoneva E. (2025). ROMO1: A Distinct Mitochondrial Protein with Dual Roles in Dynamics and Function. Antioxidants.

[12] Tsoneva E., Dimitrova P.D., Metodiev M., Shivarov V., Vasileva-Slaveva M., Yordanov A. (2023). The effects of ROMO1 on cervical cancer progression. Pathol. Res. Pract..

[13] Kim H.J., Jo M.J., Kim B.R., Kim J.L., Jeong Y.A., Na Y.J., Park S.H., Lee S.Y., Lee D.H., Lee H.S. (2017). Reactive oxygen species modulator-1 (Romo1) predicts unfavorable prognosis in colorectal cancer patients. PLoS ONE.

[14] Chung J.S., Park S., Park S.H., Park E.R., Cha P.H., Kim B.Y., Chung Y.M., Woo S.R., Han C.J., Kim S.B. (2012). Overexpression of Romo1 promotes production of reactive oxygen species and invasiveness of hepatic tumor cells. Gastroenterology.

[15] Swarnabala S., Gattu M., Perry B., Cho Y., Lockey R.F., Kolliputi N. (2014). ROMO1 links oxidative stress to mitochondrial integrity. J. Cell Commun. Signal..

[16] Zhou D., Sun M.H., Lee S.H., Cui X.S. (2021). ROMO1 is required for mitochondrial metabolism during preimplantation embryo development in pigs. Cell Div..

[17] Na A.R., Chung Y.M., Lee S.B., Park S.H., Lee M.S., Yoo Y.D. (2008). A critical role for Romo1-derived ROS in cell proliferation. Biochem. Biophys. Res. Commun..

[18] Saxena A., Carninci P. (2011). Long non-coding RNA modifies chromatin: Epigenetic silencing by long non-coding RNAs. Bioessays.

[19] Guil S., Darzynkiewicz E., Bach-Elias M. (2002). Study of the 2719 mutant of the c-H-ras oncogene in a bi-intronic alternative splicing system. Oncogene.

[20] Huang S.M., McCance D.J. (2002). Down regulation of the interleukin-8 promoter by human papillomavirus type 16 E6 and E7 through effects on CREB binding protein/p300 and P/CAF. J Virol..

[21] Kashatus J.A., Nascimento A., Myers L.J., Sher A., Byrne F.L., Hoehn K.L., Counter C.M., Kashatus D.F. (2015). Erk2 phosphorylation of Drp1 promotes mitochondrial fission and MAPK-driven tumor growth. Mol. Cell..

[22] Rehman J., Zhang H.J., Toth P.T., Zhang Y., Marsboom G., Hong Z., Salgia R., Husain A.N., Wietholt C., Archer S.L. (2012). Inhibition of mitochondrial fission prevents cell cycle progression in lung cancer. FASEB J..

[23] Chen H., Chomyn A., Chan D.C. (2005). Disruption of fusion results in mitochondrial heterogeneity and dysfunction. J. Biol. Chem..

[24] Trachootham D., Alexandre J., Huang P. (2009). Targeting cancer cells by ROS-mediated mechanisms: A radical therapeutic approach?. Nat. Rev. Drug Discov..

[25] Sabharwal S.S., Schumacker P.T. (2014). Mitochondrial ROS in cancer: Initiators, amplifiers or an Achilles’ heel?. Nat. Rev. Cancer.

[26] Buzzai M., Bauer D.E., Jones R.G., Deberardinis R.J., Hatzivassiliou G., Elstrom R.L., Thompson C.B. (2005). The glucose dependence of Akt-transformed cells can be reversed by pharmacologic activation of fatty acid beta-oxidation. Oncogene.

[27] Vazquez A., Kamphorst J.J., Markert E.K., Schug Z.T., Tardito S., Gottlieb E. (2016). Cancer metabolism at a glance. J. Cell Sci..

[28] Li N., Chamkha I., Verma G., Swoboda S., Lindstedt M., Greiff L., Elmér E., Ehinger J. (2024). Human papillomavirus-associated head and neck squamous cell carcinoma cells rely on glycolysis and display reduced oxidative phosphorylation. Front. Oncol..

[29] Bordt E.A., Clerc P., Roelofs B.A., Saladino A.J., Tretter L., Adam-Vizi V., Cherok E., Khalil A., Yadava N., Ge S.X. (2017). The Putative Drp1 Inhibitor mdivi-1 Is a Reversible Mitochondrial Complex I Inhibitor that Modulates Reactive Oxygen Species. Dev. Cell..

[30] Kirshner J.R., He S., Balasubramanyam V., Kepros J., Yang C.Y., Zhang M., Du Z., Barsoum J., Bertin J. (2008). Elesclomol induces cancer cell apoptosis through oxidative stress. Mol. Cancer Ther..

[31] Samuni Y., Goldstein S., Dean O.M., Berk M. (2013). The chemistry and biological activities of N-acetylcysteine. Biochim. Biophys. Acta.

[32] Kwok B.H., Koh B., Ndubuisi M.I., Elofsson M., Crews C.M. (2001). The anti-inflammatory natural product parthenolide from the medicinal herb Feverfew directly binds to and inhibits IkappaB kinase. Chem. Biol..

